# Amino acid metabolites, mTORC1 and aging

**DOI:** 10.18632/aging.101266

**Published:** 2017-07-17

**Authors:** Henri Antikainen, Khoosheh Khayati, Radek Dobrowolski

**Affiliations:** Federated Department of Biological Sciences, Rutgers University/New Jersey Institute of Technology, Newark, NJ 07102, USA

**Keywords:** mTORC1, autophagy, aging, amino acid, metabolites

Dysregulated metabolic signaling leading to increased mechanistic target of rapamycin complex 1 (mTORC1) activity has been associated with cancer, neuro-degeneration, and an acceleration of the aging process [1]. Aging is characterized by a systemic decline of metabolic processes, including amino acid metabolism, that results in a steady accumulation of the non-proteinogenic amino acid homocysteine (Hcy). Hcy is a metabolite of the essential amino acid methionine, and is metabolized to cysteine in a vitamin B6-dependent transsulfuration pathway catalyzed by cystathionine beta synthase (CBS), or back into methionine through vitamin B12- and folate-dependent re-methylation. Deficiencies in these pathways cause hyperhomo-cysteinemia (HHcy) and are linked to cardiovascular and brain pathologies, including age-related sporadic Alzheimer's disease (AD). Hcy might contribute to the AD pathophysiology by either directly inducing or aggravating endothelial dysfunction leading to cerebral microangiopathy (small vessel disease) and neuro-toxicity by triggering oxidative stress and neuro-inflammation. A number of pathways have been associated with Hcy-induced cytotoxicity, including the activation of neuronal NMDA receptors as well as transcriptional regulation of β- and γ-secretases that drives amyloidosis.

We have previously shown that Hcy, but not its direct metabolites such as Hcy-thiolactone and cysteine, upregulates the activity of mTORC1, one of the major metabolic kinases in cells [2]. Mechanistically, Hcy binds with relatively low affinity to leucyl-tRNA synthetase (LeuRS), an enzyme known for charging cognate tRNAs for protein biosynthesis. LeuRS maintains accurate translation by editing the noncognate Hcy and regulates the activity of the mTOR-activator Folliculin in a Hcy/Leucine-dependent manner [2]. It is important to state that another cellular leucine sensor, a protein strongly associated with aging, called Sestrin [3,4] may be implicated in sensing Hcy and Hcy-related amino acids. The role of Sestrin2 in sensing amino acid metabolites needs to be addressed in future studies. While Sestrin2 protein levels were largely unaltered in Cbs-deficient mice (a mouse model showing chronically high Hcy levels as well as a subset of AD hallmarks), it is likely that deficiency in Sestrin2 is implicated in very early events of AD pathology, rendering cells insensitive to amino acid starvation and unable to reduce activity of their mTORC1 [5].

Chronically high mTORC1 deregulates neuronal clearance and autophagy pathways leading to accumulation of aggregation prone proteins such as phospho-Tau and beta-Amyloid. Aggregation of these protein aggregates is widely discussed to drive AD-like spongiform neurodegeneration. Indeed, it suggests itself that Hcy-mediated effects on cellular metabolism through mTORC1 are not only caused by autophagy inhibition, but also by increased protein and lipid synthesis, resulting together in steadily increasing levels of reactive oxygen species, endoplasmic reticulum stress, unfolded protein response, and eventually cell death. In general, a reduction in mRNA translation using mTOR inhibitors is associated with a reduction of proteotoxic and oxidative stress that extends lifespan [6]. Additionally, chronically elevated mTORC1 activity inhibits autophagic clearance leading to an accumulation of cytotoxic proteins and defective organelles, such as depolarized mitochondria which are directly linked to programmed cell death. Several lines of evidence support the notion that general inhibition of mTOR prevents age-related diseases, including AD, and extends lifespan in experimental model systems. However, the commonly used mTOR inhibitors show severe side effects by inducing glucose intolerance and suppressing immune response. The reason for these undesired side effects is the limited selectivity of these drugs resulting in an inhibition of mTORC2. Clearly, there is a need for development of new small inhibitors able to specifically target mTORC1; hopes are high for the mTORC1-specific amino acid sensing pathways as targets for such inhibitors. A promising group of naturally occurring compounds affecting these path-ways are amino acid metabolites that can bind to cellular sensors to regulate mTORC1 without affecting mTORC2 activity. One of these metabolites, the leucine derivative leucinol, prevents dissociation of the LeuRS-Flcn complex (one of the cellular leucine sensors) from, and binding of mTORC1 to lysosomal membranes [2]. While these results are innovative, provocative and promising at the same time, a direct assessment of selectivity and specificity of these amino acid metabolites as well as an assessment of their effects on longevity will need to be addressed in future studies.
